# Bioinformatics Resource Manager: a systems biology web tool for microRNA and omics data integration

**DOI:** 10.1186/s12859-019-2805-6

**Published:** 2019-05-17

**Authors:** Joseph Brown, Aaron R. Phillips, David A. Lewis, Michael-Andres Mans, Yvonne Chang, Robert L. Tanguay, Elena S. Peterson, Katrina M. Waters, Susan C. Tilton

**Affiliations:** 1 Biological Sciences Division, Pacific Northwest National Laboratory, Richland, WA USA; 2 Computing & Analytics Division, Pacific Northwest National Laboratory, Richland, WA USA; 3 Environmental and Molecular Toxicology Department, Oregon State University, Corvallis, OR USA; 4 Superfund Research Center, Oregon State University, Corvallis, OR USA; 5 Present address: Department of Human Genetics, University of Utah, Salt Lake City, UT 84105 USA

**Keywords:** Bioinformatics, MicroRNA, Systems biology, Genomics, Zebrafish

## Abstract

**Background:**

The Bioinformatics Resource Manager (BRM) is a web-based tool developed to facilitate identifier conversion and data integration for *Homo sapiens* (human), *Mus musculus* (mouse), *Rattus norvegicus* (rat), *Danio rerio* (zebrafish), and *Macaca mulatta* (macaque), as well as perform orthologous conversions among the supported species. In addition to providing a robust means of identifier conversion, BRM also incorporates a suite of microRNA (miRNA)-target databases upon which to query target genes or to perform reverse target lookups using gene identifiers.

**Results:**

BRM has the capability to perform cross-species identifier lookups across common identifier types, directly integrate datasets across platform or species by performing identifier retrievals in the background, and retrieve miRNA targets from multiple databases simultaneously and integrate the resulting gene targets with experimental mRNA data. Here we use workflows provided in BRM to integrate RNA sequencing data across species to identify common biomarkers of exposure after treatment of human lung cells and zebrafish to benzo[*a*]pyrene (BAP). We further use the miRNA Target workflow to experimentally determine the role of miRNAs as regulators of BAP toxicity and identify the predicted functional consequences of miRNA-target regulation in our system. The output from BRM can easily and directly be uploaded to freely available visualization tools for further analysis. From these examples, we were able to identify an important role for several miRNAs as potential regulators of BAP toxicity in human lung cells associated with cell migration, cell communication, cell junction assembly and regulation of cell death.

**Conclusions:**

Overall, BRM provides bioinformatics tools to assist biologists having minimal programming skills with analysis and integration of high-content omics’ data from various transcriptomic and proteomic platforms. BRM workflows were developed in Java and other open-source technologies and are served publicly using Apache Tomcat at https://cbb.pnnl.gov/brm/.

**Electronic supplementary material:**

The online version of this article (10.1186/s12859-019-2805-6) contains supplementary material, which is available to authorized users.

## Background

There is an increasing need for bioinformatics tools to assist biologists having minimal programming skills with analysis and integration of high-content omics’ data from various transcriptomic and proteomic platforms. The Bioinformatics Resource Manager (BRM) is a web-based tool developed to facilitate identifier conversion and data integration for *Homo sapiens* (human), *Mus musculus* (mouse), *Rattus norvegicus* (rat), *Danio rerio* (zebrafish), and *Macaca mulatta* (macaque), as well as perform orthologous conversions among the supported species. BRM is particularly focused on reducing data fragmentation throughout these processes, allowing users to upload full tables of data, then appending new columns directly into those tables or directly integrating full tables based on common (or converted) identifiers.

Biological insight relies on the interpretation of annotated data. Often annotations need to be converted from one identifier to another or carried over to an orthologous annotation for some downstream tasks. DAVID [1] provides functionality for converting identifiers within a species but lacks the ability to look up orthologous genes. BioMart [2] integrates internal and external data to convert identifiers and provide orthologous gene information for model organisms. The functionality of these web-based conversion tools, like BRM, relies on user provided gene lists, although DAVID and BioMart lack the ability to merge identifier conversions with existing datasets. BRM also allows users to integrate data tables based on (1) string matching for tables that include common identifier types or (2) identifier conversion using National Center for Biotechnology Information (NCBI), Uniprot and Ensembl databases to allow for integration of tables without common identifier types (e.g. cross-species integration, gene-to-protein integration). Other tools, such as GeneWeaver, allow for identifier mapping within the context of their data analysis pipeline and tools for functional genomics [3]. While BRM will also perform these functions within the context of BRM workflows, it allows users to simply update their omics tables with new metadata and biomolecular identifiers for use in any data analysis or software programs of interest.

In addition to providing a robust means of identifier conversion, BRM also incorporates a suite of microRNA (miRNA)-target databases upon which to query target genes or to perform reverse target lookups using gene identifiers. MiRNAs are small ~ 22 nucleotide non-coding RNAs that function as post-transcriptional regulators of gene expression. miRNAs typically interact with targets through sequence complementarity in the 3’UTR making it possible to computationally predict miRNA gene targets. Several tools exist to link miRNAs to gene targets, including both computationally predicted miRNA target databases and databases with experimentally validated targets (reviewed by Singh 2017). Available databases in BRM for miRNA target prediction include TargetScan [4], microRNA.org [5], and MicroCosm [6], as well as the validated miRNA target database miRTarBase [7]. Each of these databases also allow searching for miRNA targets and performing reverse target queries based on gene ID. However, for input, many existing miRNA database interfaces are limited to single miRNA queries with the exception of microRNA.org which allows a comma-separated list of multiple identifiers. Further, the user will again have to perform table merges to align respective miRNAs into their gene result tables. Where miRNA names are inconsistent, a user may have to use miRBase [8] to verify conversions or use a dedicated tool like miRiadne [9] to convert miRNA identifiers between miRBase versions 10 through 21. Instead, BRM allows users to integrate predicted targets from databases directly into the experimental tables they have uploaded into BRM as input. BRM also integrates miRBase versions to convert user miRNAs to their most recent version before querying miRNA databases to ensure successful searches.

The BRM miRNA-target query allows users to retrieve targets from multiple databases simultaneously and integrate the resulting gene targets with experimental mRNA data. By utilizing multiple databases, a single search not only yields results from all available databases, it also allows a user to select more confident predictions by requiring targets to be present in multiple databases. Other available tools, such as miDIP 4.1, allow for simultaneous query of multiple miRNA target databases for human only [10] or provide users with the ability to integrate predicted targets from a single database with mRNA data, such as miRTrail [11]. In addition, BRM’s miRNA workflows populate missing identifier fields that are typically created from merging multiple target identification resources providing users with more comprehensive output to accurately compare across multiple prediction tools.

## Construction and content

BRM is a web application implemented in Java and Extensible Hypertext Markup Language. The front-end of BRM relies on PrimeFaces, an implementation of the Java Server Faces specification, to build user interface components. Data sources are maintained as flat files to facilitate database updates and are stored in memory during runtime to accelerate ID conversion and lookups across data resources to make BRM responsive even with fairly large user queries. BRM has been developed as an independent web tool, compared to utilizing platforms for tool development such as Galaxy [12], to allow flexibility to meet specific development requirements and maintain a straightforward, easy-to-use interface for the biological research community. BRM allows users to upload data directly into a simple web interface and provides several comprehensive workflows, which users can run independently for specific tasks or sequentially to allow users to seamlessly move data through multiple tasks. Maintaining BRM in this way allows us to optimize functionality and ensure consistency for users over time. Further, BRM is easily extended by its developers and has the ability to scale beyond the current data to accommodate additional tools, functionality, biomolecular identifiers and species.

BRM maintains local copies of NCBI’s Gene resource [13], Ensembl [14], and UniProt [15] for identifier conversions. MiRNA reference data is aggregated from Microcosm, TargetScan, MicroRNA, and miRTarBase with missing gene information being added using MyGene.info [16]. miRbase is used for miRNA name conversion, accession numbers, and mature sequence data. Each data resource has an associated backup process that facilitates validation, database updates, and to backfill missing identifiers across resources.

## Utility and discussion

### Overview

BRM incorporates common tasks across highly relevant species to facilitate the integration and analysis of high-throughput data. The BRM web tool is organized into several workflows, 1) Add Identifiers, 2) Integrate Tables, 3) miRNA Targets and 4) miRNA Convert, allowing biological researchers the ability to perform complex bioinformatics tasks through a simple web-interface. Users can retrieve annotations and cross-reference gene and protein identifiers for several species, including human, macaque, mouse, rat and zebrafish and identify miRNA targets for human, mouse and zebrafish. Further, BRM allows datasets to be uploaded as tab-separated (.txt) files with columns in any order and will maintain the structure and content of user-provided data during queries. This allows users to easily incorporate additional content into their datasets to perform comparisons across species and platforms (e.g. transcriptomics and proteomics; microarray and RNA sequencing (RNASeq); in vitro and in vivo). BRM also provides a tool for directly integrating datasets across platform or species by performing identifier retrievals in the background. The BRM ‘miRNA Targets’ and ‘miRNA Convert’ workflows allow users to quickly identify miRNA gene targets from multiple databases, integrate miRNA and mRNA datasets based on target predictions, and retrieve current miRNA annotations for metadata from older platforms.

### Cross-species identifier

**Fig. 1:**
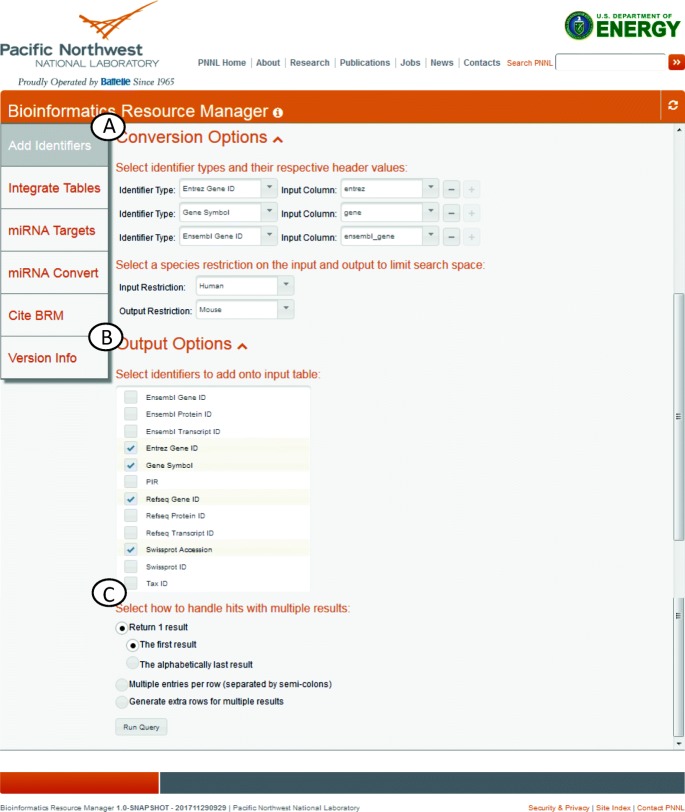
BRM Cross-Species Identifier Query. BRM performs cross-species identifier lookups across common identifier types such as Ensembl, Entrez, and gene symbol, and performs orthologous lookups using Ensembl as the common identifier. (**a**) After uploading, the user defines columns and column types, e.g. Entrez Gene ID, using dropdown selection boxes. Up to three identifiers can be used per data entry to ensure successful conversion. (**b**) Users then select the identifiers to add onto the input Table. (**c**) Then, the user chooses how to handle entries with multiple hits. By default, the first result is returned or users can select to allow multiple entries per row or multiple rows per result

BRM performs cross-species identifier lookups across common identifier types such as Ensembl, Entrez, and gene symbol, and performs orthologous lookups using Ensembl as the common identifier (Fig. 1). User input for this tool is a tab-delimited text file containing a header. After uploading, the user defines columns and column types, e.g. Entrez Gene ID, using dropdown selection boxes. Up to three identifiers can be used per data entry to ensure successful conversion (Fig. 1a). All IDs in BRM’s database maintain their taxonomy ID allowing the user to separately define species restrictions for the input and output data. Without any output restriction, all orthologous hits will be returned. After selecting the types of IDs to append onto the data (Fig. 1b), the user has a choice in how to handle entries with multiple hits (Fig. 1c). By default, the first result is returned though options allow multiple entries per row or multiple rows per result.

### Data integration

This tool integrates disparate data tables based on identifiers contained within the tables uploaded. Users have the ability to integrate data across species or platform (e.g. gene and protein data) without common identifiers in the tables. After uploading data, the user may select up to three identifier columns from each table upon which to perform the merge operation. Identifiers between tables can be compared using string equality, which performs a simple exact match, or conversions of identifiers within or across species can be performed. The output from this tool can be limited to a particular species as well as limited to just the intersection of the two input tables. Another important aspect of the data integration tool is that all user-provided data is maintained in the merge and the output includes a full integration of both tables based on the features chosen (see example in Cross-Species Data Integration below).

### miRNA target prediction

Predicted gene targets from Microcosm, MicroRNA, and TargetScan, as well as experimentally validated gene targets from miRTarBase, can be queried using mature miRNA names. Mature miRNA names are converted to their current miRBase name during the search process. Target genes include identifiers for Entrez, gene symbol, and Ensembl gene and can optionally be appended to miRNA target prediction results. Gene target results can be limited to any combination of the databases and can be limited based on database overlap, e.g. require hits from at least 2 of the 4 selected databases. The workflow can optionally merge experimental data based on gene identifiers that match the predicted targets. Results include gene targets, database overlaps, respective scores from predictive databases, accession numbers for the stem-loop and mature miRNA, and the mature RNA sequence.

A reverse lookup, starting from gene identifiers as targets, can also be performed to return mature miRNA names. Multiple gene ID types may be used from the input table to ensure successful translation.

### miRNA conversions

To facilitate analyses across tools it may necessary to convert miRNA identifiers to their most current miRBase version. This workflow, given a tab-delimited table, will accept one column as the defined miRNA and append its most recent version as the final column in the output. The output and conversion of identifiers can be restricted to a given species.

### Cross-species data integration

**Fig. 2:**
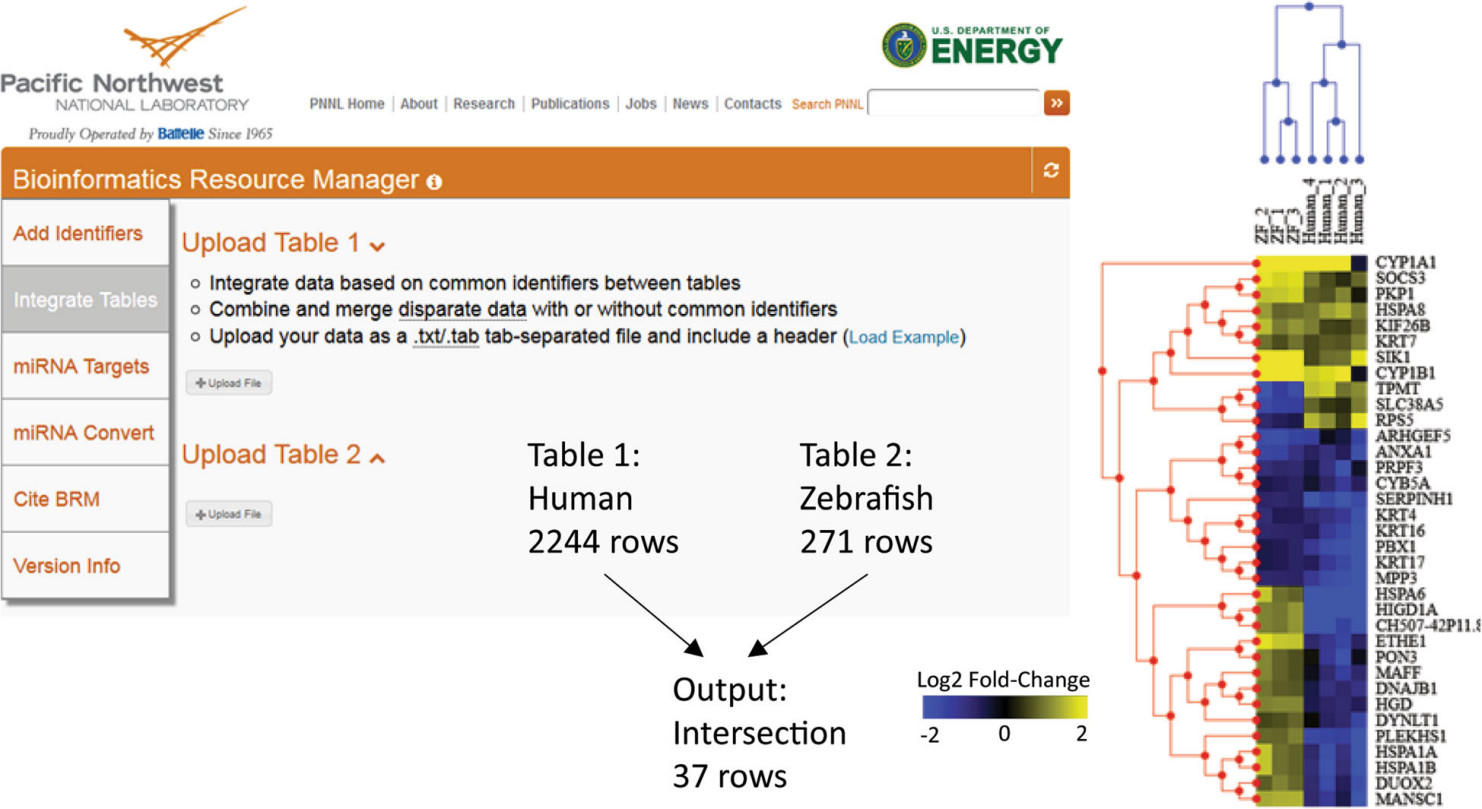
BRM Cross-Species Data Integration. The Integrate Tables workflow in BRM was utilized to integrate global transcriptomics data collected from human bronchial epithelial cells and zebrafish embryos after exposure to benzo[*a*]pyrene (BAP) for 48 h. Datasets were integrated based on Ensembl Gene ID for each species resulting in the intersection of 37 genes between datasets, which were visualized as a clustering heatmap to evaluate similarity in gene expression (Log2 fold-change) between species

Here we present an example in which global transcriptomics analyses from two species are integrated in BRM to identify the subset of genes regulated in common after exposure of zebrafish embryos and human bronchial epithelial cells (HBEC) exposed to benzo[*a*]pyrene (BAP) for 48 h. BAP is a ubiquitous contaminant in the environment from the incomplete combustion of fossil fuels from sources such as cigarette smoke, diesel exhaust and coal tar. The data tables were uploaded as tab delimited (.txt) files into the BRM Integrate Tables feature and merged using the Ensembl Gene ID for each species. BRM performs cross-identifier conversions automatically between tables and the intersection (common entities between both datasets) were downloaded for evaluation. Exposure of HBEC cultured at the air-liquid interface to 500 μg/mL (19.8 nmol) BAP (Additional file 1) resulted in differential regulation of 2244 significant (q < 0.05) genes (Additional file 2) while exposure of zebrafish embryos to 10 uM (20 nmol) BAP [17] resulted in regulation of 271 significant (q < 0.05) genes (Additional file 3). Integration of these datasets in BRM is summarized in Fig. 2 and resulted in 37 rows in the output (Additional file 4). The integrated data were imported into WebMeV software for visualization as a clustering heatmap [18]. Overall, we can see that few genes are significantly regulated in common by BAP in human and zebrafish based on experimental parameters (described in Additional file 1) and that 50% of the genes significantly regulated by BAP in both species are oppositely expressed compared to control samples. However, transcripts for enzymes cytochrome P450 1A and 1B, which are involved in metabolism of BAP, were significantly induced after treatment in both species and serve as a common biomarker of BAP exposure. BRM provides a simple web-interface for integrating data tables in a single step.

### miRNA target prediction and data integration

In order to identify miRNAs predicted to regulate genes significantly altered by BAP exposure in human cells, we utilized the reverse look-up feature (gene-to-miRNA query) of the miRNA Targets workflow in BRM. A tab delimited (.txt) file of genes differentially expressed (q < 0.05) by BAP in HBEC were uploaded to the miRNA Targets workflow (Additional file 2). Predicted miRNAs were restricted to those that were identified from any 4 of 4 target databases, meaning that the miRNA-gene target relationship was predicted by all data sources, including Microcosm, MicroRNA, TargetScan and miRTarBase. The miRNA predicted from this analysis associated with the most target interactions in the dataset was hsa-miR-124-3p, which was connected to 27 gene targets regulated by BAP. MiRNA-124-3p was recently found to be overexpressed in smokers at increased risk of cardiovascular disease [19] and elevated in HepaRG cells after BAP exposure [20].

**Fig. 3:**
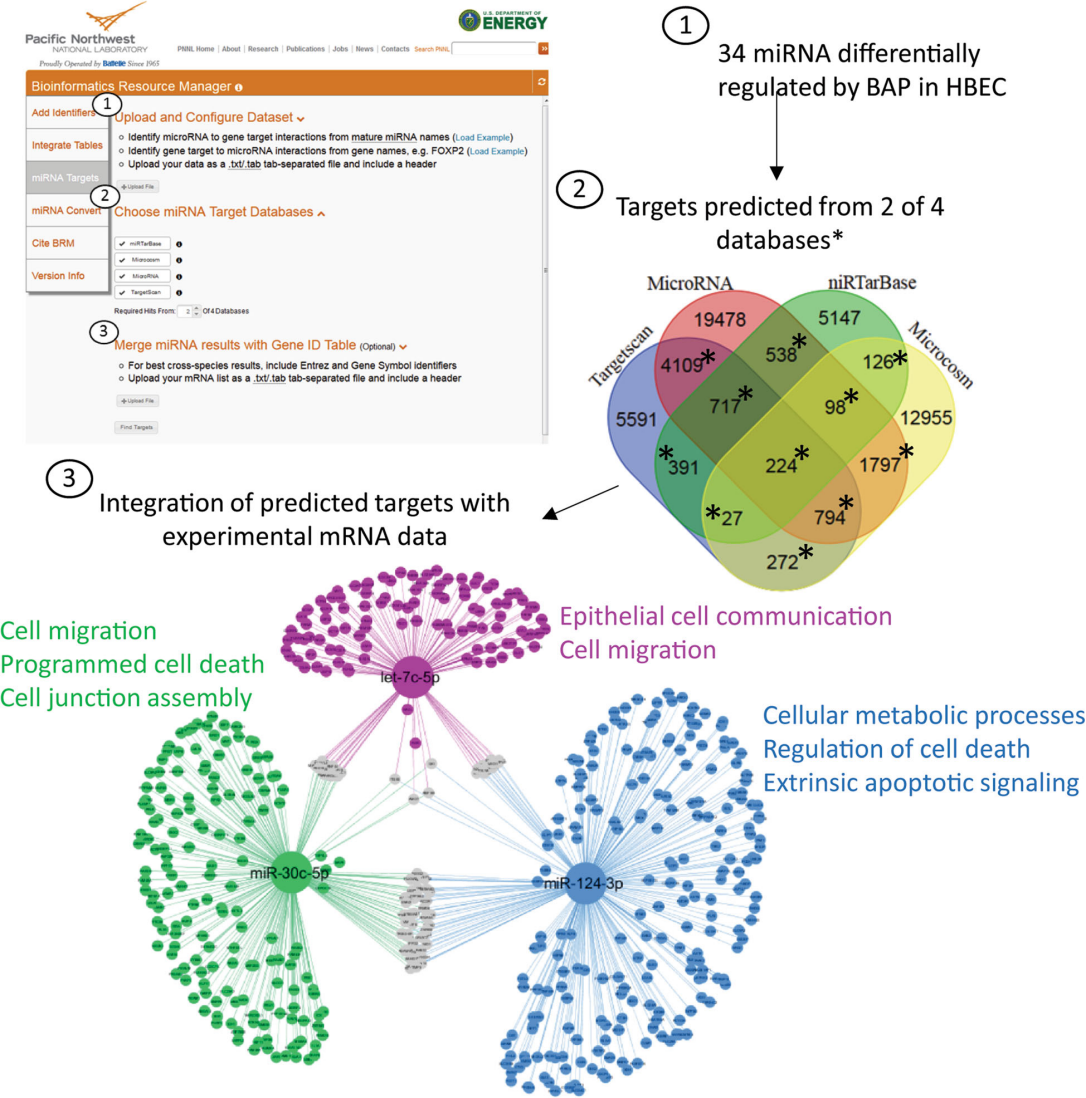
miRNA Target Prediction and Integration Workflow. The miRNA Targets query was utilized to (1) upload a list of 32 significant (q < 0.05) miRNA differentially expressed in human bronchial epithelial cells (HBEC) after exposure to benzo[*a*]pyrene (BAP), (2) identify potential miRNA gene targets from Microcosm, MicroRNA, TargetScan and miRTarBase resources, filtering for targets that are in at least 2 of the 4 databases, and (3) integrate the predicted gene targets with mRNA expression data collected in parallel in HBEC. The resulting miRNA-target gene interactions for the 3 most connected miRNAs are visualized as a network with significantly (*p* < 0.05) enriched biological function GO terms included for each subnetwork

To experimentally determine the role of miRNAs as regulators of BAP toxicity, miRNAs were measured in parallel with mRNA in HBEC after exposure to 500 μg/ml (19.8 nmol) BAP for 48 h by RNAseq. Overall, a total of 32 miRNAs were significantly (q < 0.05) regulated by BAP in HBEC, including miR-124-3p which was predicted through the reverse look-up above. This dataset was uploaded to the miRNA Targets workflow in BRM as a tab delimited (.txt) file using the miRNA-to-gene query type to identify predicted targets of miRNAs regulated by BAP in human lung cells (Additional file 5, Fig. 3, step 1). Overall, 52,264 unique miRNA-target interactions were predicted in human for all 32 miRNA. In order to increase confidence of target predictions and reduce the potential for false positives, target interactions were limited to only those predicted by at least 2 of the 4 data sources, which resulted in 9093 unique miRNA-target interactions in the target query output (Additional file 6, Fig. 3, step 2). The optional ‘Merge miRNA results with Gene ID Table’ feature was utilized to integrate predicated targets with experimental mRNA collected in parallel from HBEC after BAP exposure (Additional file 2, Fig. 3, step 3). Out of the 2244 genes significantly altered by BAP treatment in HBEC, 835 genes overlapped with predicted gene targets identified in the BRM miRNA Targets workflow. MiRNA-gene interactions were visualized in Cytoscape [21] for the 3 largest subnetworks (miR-let-7c-5p, miR-30c-5p and miR-124-3p). The genes in each subnetwork were analyzed for significantly enriched functional processes using the DAVID Bioinformatics Functional Annotation tools [1] and example processes (*p* < 0.05) are shown (Fig. 3). Overall, these data show a role for miRNAs as potential regulators of BAP toxicity in HBEC associated with cell migration, cell communication, cell junction assembly and regulation of cell death. Similar functional roles for these miRNAs have previously been reported in human cancer cells [22–24].

## Conclusions

BRM provides easy to follow workflows to assist biological researchers with complex bioinformatics tasks required for integration of disparate data types (e.g. cross-species and cross-platform) with specific tools for miRNA target prediction and conversion. Previous versions of the BRM software provided similar tools in a client-server application [25, 26], however compatibility with multiple operating systems (Windows vs Mac) and evolving support software (java runtime environment) resulted in several versions to support and maintain. In this new version, we have converted several of the old tools, such as the identifier conversion and miRNA target query, into seamless web interfaces without the need to download software or remember login information. We have also updated the workflows to simplify multiple steps through identifier conversions that happen in the background. Here, we provide example datasets and workflows for utilizing the BRM data integration tool to identify common biomarkers in humans and zebrafish after exposure to a ubiquitous environmental contaminant, BAP. BRM integrated the two RNAseq data tables from human and zebrafish utilizing the cross-species functionality without requiring any common identifiers. Further, BRM maintained the content and structure of the uploaded files during the integration for direct use in downstream visualization tools for interpretation. The BRM miRNA Targets workflow was also utilized to identify the potential functional consequences of miRNA regulation by BAP in human lung cells and involved target prediction of experimentally measured miRNAs and integration of predicted targets with differentially expressed mRNA collected in parallel. The resulting output included a list of high-confidence predicted targets for miRNAs regulated by BAP that were relevant to our experimental system and directly uploaded into other freely available software tools for additional analysis. Overall, BRM allows for efficient processing and integration of multiple data types within a single tool and provides users the ability to effectively mine complex data.

## Additional files

Additional file 1: Experimental Methods. Description of experimental methods for datasets used in the paper, including culturing, treatment protocols, RNA sequencing and data analysis for HBEC and zebrafish embryos. (PDF 14 kb)

Additional file 2: HBEC mRNA list. List of differentially expressed mRNA in HBEC after treatment with BAP. (TXT 336 kb)

Additional file 3: Zebrafish mRNA list. List of differentially expressed mRNA in zebrafish after treatment with BAP. (TXT 33 kb)

Additional file 4: Human-zebrafish integration BRM output. Output from BRM after integrating human and zebrafish mRNA files using the Integrate Tables feature. (XLSX 19 kb)

Additional file 5: HBEC miRNA list. List of differentially expressed miRNA in HBEC after treatment with BAP. (TXT 953 bytes)

Additional file 6: Zebrafish miRNA list. List of differentially expressed miRNA in zebrafish after treatment with BAP. (TXT 1305 kb)

## Abbreviations

BAP: Benzo[*a*]pyrene
BRM: Bioinformatics resource manager
HBEC: Human bronchial epithelial cells
miRNA: MicroRNA
NCBI: National center for biotechnology information
RNAseq: RNA sequencing
