# Supplementary material for: Bioinformatics Resource Manager: a systems biology web tool for microRNA and omics data integration
Source: BMC Bioinformatics. 2019 May 17;20:255. doi: 10.1186/s12859-019-2805-6 (PMC6525352; doi:10.1186/s12859-019-2805-6)
Supplement: Supplementary file 1 — Experimental Methods. Description of experimental methods for datasets used in the paper, including culturing, treatment protocols, RNA sequencing and data analysis for HBEC and zebrafish embryos. (PDF 14 kb) [file 12859_2019_2805_MOESM1_ESM.pdf]

Additional File 1. Experimental Methods

Human bronchial epithelial cell (HBEC) Culture and Treatments. EpiAirway™ 100 tissues were shipped overnight and received chilled on ice packs. Tissues were immediately transferred to 6-well plates each well containing 1 ml of assay medium and equilibrated for 24 hours at 37°C, 5% CO_2 followed by a change of fresh medium before any treatment regimens commenced. Initiation with PAHs was timed so that all treatments would be harvested on the same day thus all tissues would be cultured for the same number of days. Tissues were prepared for treatment by rinsing the apical surface of the inserts once with 0.50 ml PBS and carefully aspirating PBS to remove mucus from the surface of the tissues. The medium was replaced with 1 ml of fresh medium. Benzo[a]pyrene (BAP; CAS# 50-32-8, MRIGlobal, Kansas City, MO) was added to the apical surface of tissues in 5 ul acetone using a pipette. After 48 hours, cells were placed in cryovials containing 0.5ml TRIzol® reagent (ThermoFisher Scientific) and snap frozen in liquid nitrogen. Frozen cells were stored at -80°C for RNA isolation and sequencing. RNA was isolated following TRIzol Reagent manufacturer's protocol. mRNA was poly-A selected, and libraries were prepared with the PrepXTM mRNA and Illumina sequencing workflow (Wafergen Biosystems, Fremont, CA). Paired-end sequencing (150 bp) was conducted with an Illumina HiSeq 3000 sequencer.

Zebrafish care, husbandry and BAP exposure: Adult Tropical 5D strain zebrafish (*Danio rerio*) were raised according to Institutional Animal Care and Use Committee protocols in the Sinnhuber Aquatic Research Laboratory at Oregon State University. Adults were maintained on a 14 h light/10 h dark schedule on a recirculating system in which water was maintained at 28±1 °C with a pH of 7.0±0.2. Zebrafish embryos were exposed to 10 uM (20 nmol) BAP in 0.1% DMSO beginning at 6 hpf. Whole embryos were collected for RNA isolation at 48 hpf and sequenced by paired-end mRNAseq using Illumina HiSeq 2000 as described previously[1].

RNAseq processing and data analysis: RNAseq analysis was performed using the Tuxedo suite pipeline. Tophat version 2.1.1[2] aligned paired-end sequence reads to the Danio rerio genome assembly Zv9 or the Homo Sapiens genome assembly GRCh38.84. Transcripts were assembled using Cufflinks v 2.2.1 and merged using Cuffmerge v. 1.0.0 using the Ensembl genomes to guide the assembly[3]. Analysis of differential expression was performed with CuffDiff to identify the significant transcript expression for each exposure group compared to control. Raw files are available in GEO (GSE128471 and GSE128471).

References:

1. Goodale BC, La Du J, Tilton SC, Sullivan CM, Bisson WH, Waters KM, Tanguay RL. Ligand-Specific Transcriptional Mechanisms Underlie Aryl Hydrocarbon Receptor-Mediated Developmental Toxicity of Oxygenated PAHs. Toxicological sciences : an official journal of the Society of Toxicology. 2015;147(2):397-411. Epub 2015/07/05. doi: 10.1093/toxsci/kfv139. PubMed PMID: 26141390; PMCID: PMC4612346.
2. Trapnell C, Pachter L, Salzberg SL. TopHat: discovering splice junctions with RNA-Seq. Bioinformatics (Oxford, England). 2009;25(9):1105-11. Epub 2009/03/18. doi: 10.1093/bioinformatics/btp120. PubMed PMID: 19289445; PMCID: PMC2672628.
3. Trapnell C, Hendrickson DG, Sauvageau M, Goff L, Rinn JL, Pachter L. Differential analysis of gene regulation at transcript resolution with RNA-seq. Nature biotechnology. 2013;31(1):46-53. Epub 2012/12/12. doi: 10.1038/nbt.2450. PubMed PMID: 23222703; PMCID: PMC3869392.
